# Real World Data for Pancreatic Adenocarcinoma from a Population-Based Study in France

**DOI:** 10.3390/cancers15020525

**Published:** 2023-01-15

**Authors:** Bogdan Badic, Marie Morvan, Lucille Quénéhervé, Servane Bouzeloc, Tiphaine Kermarrec, Jean-Baptiste Nousbaum, Noémi Reboux

**Affiliations:** 1 Digestive Surgery Department, CHRU Brest, 29200 Brest, France; 2 Registre des Cancers Digestifs du Finistère, 29609 Brest, France; 3 LaTIM-Laboratory of Medical Information Processing, INSERM UMR 1101, Université Bretagne Occidentale, 29238 Brest, France; 4 EA7479 SPURBO, Université de Bretagne Occidentale, 29200 Brest, France; 5 CHRU Brest, Service d’Hépato-gastro-entérologie, 29200 Brest, France

**Keywords:** pancreatic adenocarcinoma, survival, surgery, chemotherapy, radiotherapy, metastasis

## Abstract

**Simple Summary:**

Pancreatic cancer is one of the most lethal digestive cancers and is becoming more common over the decades. The analysis of prognostic factors for survival could help to evaluate the existing treatments and find better treatment options. Our retrospective study analyzed data from 2117 patients issued from a digestive tumor registry. Age, surgery, chemotherapy, radiotherapy, and metastasis location are prognostic factors correlated with overall survival. Although this study is retrospective, our data were collected across multiple sites and reflect the outcomes of the management of patients with pancreatic cancer over a long period.

**Abstract:**

Pancreatic cancer is associated with high mortality rates, and most cases are diagnosed at advanced stages. This study aimed to evaluate the prognostic factors for survival in pancreatic adenocarcinoma. Data from the Finistere registry of digestive database were used in this analysis. This retrospective population-based study included 2117 patients with pancreatic adenocarcinoma diagnosed between 2005 and 2019. Cox regression was used to assess the impact of different prognostic factors. The overall median age was 74 (IQR 65.0–81.0). The majority of pancreatic adenocarcinoma 1120 (52.90%) occurred in the head of the pancreas. The type of surgical resection correlated with age (pancreaticoduodenectomy performed in 13.39% of patients aged under 65 years and only 1.49% of patients aged ≥ 80 years). For the entire cohort, 1-year mortality rate after diagnosis was 77.81%. Chemotherapy was associated with better survival for both operated (HR 0.17 95% CI 0.22; 0.64 *p* < 0.001) and unoperated patients (HR 0.41 95% CI 0.27; 0.61 *p* < 0.001). Palliative radiotherapy was associated with improved survival (HR 0.69 95% CI 0.56; 0.85 *p* < 0.001). Among operated patients, the presence of lung metastases (median 34.06; CI 20.06; 34.66) was associated with better survival compared with liver metastases (median 21.10; CI 18.10; 28.96), peritoneal carcinomatosis (median 11.00; CI 8.53; 14.63), or distant metastases (median 15.16; CI 12.66; 18.13) (*p* = 0.0001). Age, curative surgery, positive lymph nodes, chemotherapy, and palliative radiotherapy were corelated with overall survival. Surgical resection is the only potentially curative treatment, but less than a quarter of patients were eligible.

## 1. Introduction

Pancreatic cancer is a highly fatal disease, with only about 10% alive five years after the diagnosis [1]. More than half of pancreatic cancer patients are diagnosed with a metastatic or locally advanced disease [2]. It has been predicted that pancreatic cancer deaths will become the third most important cause of cancer death in the European Union, after lung and colorectal cancer [3].

Pancreatic cancer patients develop few, if any, symptoms until cancer spreads to an advanced stage, and only a minority of patients have a surgically resectable tumor. Several symptoms can indicate pancreatic cancer: jaundice (50%), change in bowel habit (40%), fatigue (50%), weight loss (55%), back pain (25%), and decreased appetite (48%) [4]. Often, more than one symptom is present in the same patient, and the tumor location is a determinant of the initial presentation. Complete surgical resection, with low perioperative mortality in specialized centers, and advantageous tumor characteristics are the most crucial factors to achieving long-term survival. However, only 20% of patients are diagnosed with pancreatic tumors in a resectable stage, and about 60% present with metastatic disease or poor performance status, thus precluding surgical removal [5]. Associated with more advanced stages, tumor size might be negatively associated with resection frequency because larger lesions are more prone to involving vessels. For the resected pancreatic tumors, significant differences in survival exist between tumors of 10 to 20 mm, 20 to 30 mm, and 30 to 40 mm (33 vs. 27 vs. 21 months, respectively) [6]. R0 resection (complete tumor removal with negative resection margins) is achieved in 40% to 72% of patients with reduction of the risk of death of 12% to 22% when comparing R0 to R1 resection [7]. Complication rates following pancreatic resection remain high, with a mortality of up to 4% in high-volume centers and morbidity of about 40% in large series [8]. The rate of lymph node metastases in resectable pancreatic cancer is high at about 70–80%, and 5-year survival rate in N+ tumors is about 17.4%. Some studies reported an increased likelihood of a margin-negative resection, negative lymph nodes, and improved overall survival after neoadjuvant therapy [9]. Adjuvant treatment led to significantly longer survival among patients with resected pancreatic cancer [10].

Pancreatic cancer patients in clinical trials are not always representative of the real-life population. The aim of this study was to determine the predictive factors for survival in a large cohort of unselected patients with pancreatic cancer.

## 2. Materials and Methods

### 2.1. Data Collection

**Figure 1:**
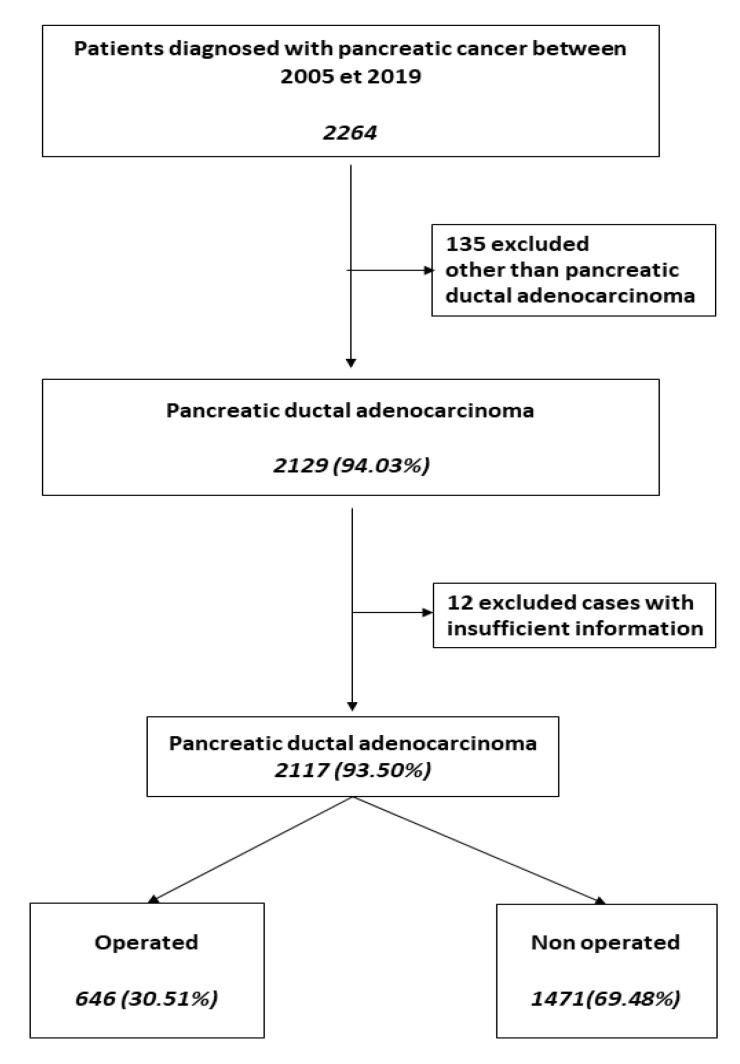
Flowchart of patients included in the study.

This study included 2117 patients diagnosed with pancreatic cancer from 1 January 2005 to 31 December 2019, which was the data cutoff for the analysis (Figure 1).

The data for this study were extracted from the Finistère digestive cancer registry database for tumor characteristics and treatments. This study focused only on pancreatic ductal adenocarcinoma. Other malignant neoplasms (acinar, neuroendocrine and colloid carcinomas, and metastases) have been excluded. Carcinomas arising from the ampulla of Vater, extrahepatic bile duct, duodenum, or stomach and involving the pancreatic parenchyma were also excluded. For all cases, information on patient demographics, tumor characteristics, pathological staging, treatment modalities, and outcomes was obtained from anatomic pathologists, general practitioners, hospitals, and private physicians (gastroenterologists, surgeons, and oncologists).

### 2.2. Statistical Analysis

The statistical analysis was performed using IBM SPSS Statistics for Windows (Version 24.0., Released 2016., Armonk, NY, USA: IBM Corp.) Qualitative data were expressed as number and frequency and quantitative data as median and range. Analysis of pancreatic cancer groups was performed by KHI2 or Fisher’s test for categorical data and Wilcoxon test for quantitative data. Overall survival (OS) was defined as the interval between the date of diagnosis and the date of death or last follow-up. Survival curves were estimated by the Kaplan–Meier method and compared using the Log Rank test. A multivariate analysis was performed using Cox proportional hazards regression model. A *p*-value < 0.05 was considered significant.

### 2.3. Ethics Approval

The quality and completeness of the Finistere Digestive cancer registry are certified every four years by an audit conducted by the National Cancer Institute (INCa), the National Institute of Health and Medical Research (INSERM), and the National Public Health Institute. The present study complied with the ethical guidelines of the 1975 Declaration of Helsinki (6th revision, 2008), our national and institutional guidelines. This observational non-interventional study was approved by the French Data Protection Authority (CNIL, authorization n° 998024).

## 3. Results

### 3.1. Patient Characteristics

**Figure 2:**
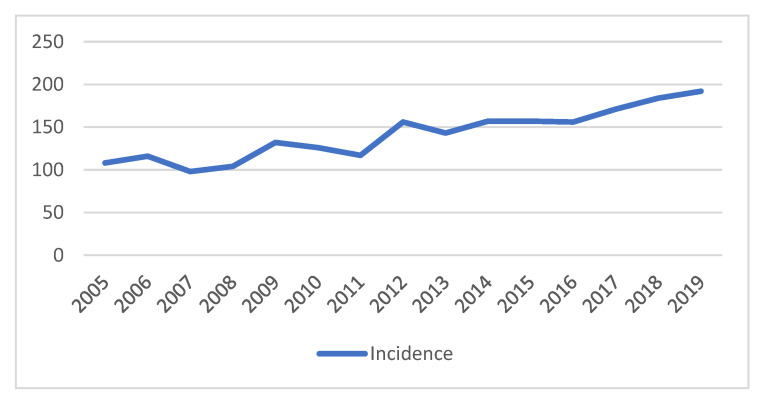
Incidence of pancreatic cancer.

Between 2005 and 2019, 2117 patients were diagnosed with pancreatic adenocarcinoma. The median age was 74 (ranging from 24 to 100 years), and 645 (30.47%) patients were 80 years or older. There were 1081 (51.06%) men, and gender was not significantly related to tumor location (*p* = 0.14). The number of pancreatic adenocarcinoma cases diagnosed each year was steadily increasing (Figure 2).

The majority of pancreatic adenocarcinoma 1120 (52.90%) occurred in the head of the pancreas, while 354 (16.72%) occurred in the body and tail. The diagnosis was revealed by suggestive symptoms such as jaundice [707 (33.39%) patients], weight loss [949 (44.82%)], nausea/vomiting [303 (13.39%)], or abdominal pain [1016 (47.99%)]. The presence of jaundice, abdominal pain, and weight loss correlated with pancreatic head tumors (*p* < 0.0001)

### 3.2. Treatment

Of the 646 (30.51%) operated patients, 41 (6.34%) patients received preoperative chemotherapy. Resection surgery was performed in 293 patients, complete resection (R0) of pancreatic tumor was realized for 200 (68.25%) patients, and 77 (26.27%) patients had R1 resection of the primary lesion (microscopic residual tumor). Pancreaticoduodenectomy was performed in 208 (70.98%) patients, distal pancreatectomy in 60 (20.47%) patients, and total pancreatectomy in 9 (3.07%). The rate of resection surgery decreased with increasing age (*p* < 0.0001); 37.79% of patients up to 65 years old underwent resection of primary tumor, while the rate decreased to 15.02% for patients aged ≥ 80 years. The type of resection also correlated with age, with pancreaticoduodenectomy performed in 13.39% of patients aged under 65 years and only 1.49% of patients aged ≥ 80 years (Table 1).

The majority of operated tumors were pT3 tumors [157 (55.47%)] with a medium size of 32 mm. Tumor size was correlated with surgical procedure (*p* < 0.0001). Lymph node involvement (as classified by the 8th edition of the AJCC system [11]) present in 65% of patients was not significantly associated with tumor location (*p* = 0.21) or tumor size (*p* = 0.30). Lymphovascular invasion was not correlated with tumor size (*p* = 0.10) or location (*p* = 0.16).

Exploratory laparotomy was realized for 353 (16.67%) patients and palliative bypass for 233 (11.0%) patients. The majority of the patients 1292 (61.02%) had metastasis at diagnosis. Liver (49.1.8%) was the main metastatic location, followed by peritoneal carcinomatosis (10.5%). Multiple site metastases were found in 423 (19.98%) of patients. Location of metastasis was correlated with primary tumor location (*p*< 0.001). Of the patients who underwent pancreatic cancer resection, 190 (67.37%) had a recurrence, including 33 (15.19%) liver recurrences, 58 (20.49%) distant metastases, and 74 (26.14%) local recurrences and distant metastases. Palliative surgery was realized for 8 (2.82%) patients with recurrence.

A total of 523 (24.75%) patients received chemotherapy, 41 (1.93%) patients were administered preoperative chemotherapy, 28 (1.32%) received intraarterial chemoinfusion, 167 (7.88%) adjuvant chemotherapy, and 320 (15.11%) palliative chemotherapy. Regardless of the type of chemotherapy intended, administration rates decreased with age (*p* = 0.001). Radiochemotherapy was delivered preoperatively to 5 (1.70%) patients and postoperatively to 11 (3.75%) resected patients. Palliative radiotherapy was given to 89 (4.87%) patients.

### 3.3. Survival and Prognostic Factors

**Figure 3:**
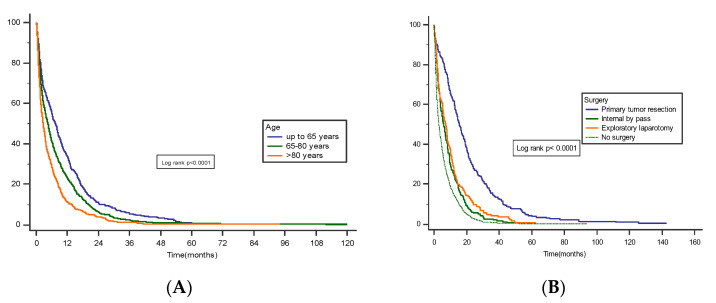
(**A**) Survival rates stratified by age groups (up to 65 years (median 7.33, 95% CI 6.20 to 8.20), 65–80 years (median 4.53, 95% CI 3.83 to 4.93), >80 years (median 2.63, 95% CI 2.23 to 3.13), Log-rank *p* < 0.0001)). (**B**) Survival rates stratified by treatment (surgery (median 15.56, 95% CI 13.50 to 18.10), internal by-pass (median 5.86, 95% CI 4.40 to 7.16), exploratory laparotomy (median 7.26, 95% CI 5.33 to 8.40), no surgery (median 3.03, 95% CI 2.70 to 3.36), Log-rank *p* < 0.0001)).

By 31 December 2019, which was the data cut-off for this analysis, 96.22% of patients had died. The median overall survival for the entire cohort was 4.2 months (IQR 42.0 to 323.50). For the entire cohort, 1-year mortality rate after diagnosis was 77.81%. The mortality rate increased with patient age (66.79% in patients younger than 65 years, 77.27% in patients 65 to 80 years, and 89.13% in patients older than 80 years, (*p* < 0.001)). The 3-year and 5-year survival rates were 6.36% (4.79% in patients younger than 65 years, 4.87% in patients aged 65 to 80 years, and 1.89% in patients older than 80 years) and, respectively, 0.7% (2.49% in patients younger than 65 years, 0.10% in patients aged 65 to 80 years, and 0% in patients older than 80 years) (Figure 3A).

Surgery was more common for pancreatic head lesions than for body and tail cancer (*p* < 0.001), but the location of the primary lesion did not correlate with better survival. Survival was higher in operated patients: median OS of 15.33 (IQR 7.90–26.63) months for patients who underwent complete resection, 6.46 (IQR 2.31–12.80) months for palliative surgery (internal bypass), and 3.03 (IQR 1.16–7.90) months for unoperated patients (*p* = 0.01). Postoperative mortality (30-day mortality) was 5.68% (Figure 3B).

Lymphovascular invasion (HR 1.44 95% CI 1.01, 2.06 *p* < 0.04), but not tumor size, correlated with overall survival in patients with resection of pancreatic cancer.

Adjuvant (HR 0.17 95% CI 0.22, 0.64 *p* < 0.001) and palliative chemotherapy (HR 0.41 95% CI 0.27, 0.61 *p* < 0.001) was associated with better survival. When comparing groups matched on age and stage but not on comorbidities or adjuvant treatment, preoperative chemotherapy or radiochemotherapy was not correlated with overall survival. Palliative radiotherapy was associated with improved survival (HR 0.69 95% CI 0.56, 0.85 *p* < 0.001). For patients with recurrence after pancreatic tumor resection, chemotherapy (HR 0.60 95% CI 0.46, 0.79 *p* < 0.001) and radiochemotherapy (HR 0.46 95% CI 0.23, 0.95 *p* = 0.03) correlated with better overall survival.

Unoperated patients with lung metastases (HR 0.67 95% CI 0.48, 0.92 *p* < 0.001) had better prognosis than those with liver and peritoneal metastases (HR 1.29 CI 1.04, 1.59). In operated patients, the presence of lung metastases (median 34.06, CI 20.06, 34.66) was associated with better survival compared to liver metastases (median 21.10, CI 18.10, 28.96), peritoneal carcinomatosis (median 11.00, CI 8.53, 14.63), or distant metastases (median 15.16, CI 12.66, 18.13) (*p* = 0.0001).

Cox univariate regression analysis showed favorable prognosis for patients with pancreatic cancer resection, well and medium differentiated pancreatic cancer, adjuvant or palliative chemotherapy, and palliative radiotherapy. Multivariate analyses revealed that age and presence of positive lymph nodes were independent poor prognostic factors for pancreatic cancer patients (Table 2).

## 4. Discussion

In our study, age and curative surgery, positive lymph nodes, chemotherapy, and palliative radiotherapy had an impact on overall survival. Surgical resection is the only potentially curative treatment, but less than a quarter of patients were eligible.

Worldwide incidence, prevalence, and mortality of pancreatic cancer increased by 55%, 63%, and 53%, respectively, during the last 25 years, accounting for 1.8% of all cancers, and 4.6% of all cancer deaths [12]. Pancreatic cancer at 40 years of age is extremely rare (2 cases per million per year), but among 80-year-old patients, the incidence is about 200 new cases per 100,000 population per year [13]. European average 5-year relative survival was 23% in the 15–44 age group, 11% in the 45–54 age group, 7% in the 55–64 age group, 5% in the 65–74 age group, and 3% for 75-year and older [14]. In our study, the 1-year mortality rate increased with patient age, with a rate of almost 90% in patients 80 years and older.

Although the overall outcome of head/uncinate cancers is better due to the high proportion of resectable cases, the poor outcome of pancreatic cancers in the body/tail is usually explained by their late detection [15]. In our series, tumors in the pancreatic tail were associated with poor prognosis. Recent large-scale genomic studies revealed aggressive tumor biology for body and tail pancreatic tumor associated with the squamous subtype [16]. Squamous tumors are associated with altered transcriptional activity of P p63, KDM6A mutations, *TGF β*, *WNT* and *ECM* signaling cascades, activated MYC, and have a poor prognosis [17]. Tumor size correlates with surgical outcomes, and pancreatic tumor diameter above 20 mm are associated with an increased rate of incomplete resection and metastatic lymph nodes [18]. In our studies, the univariate analysis found that tumor size was correlated with overall survival. Tumors less than 2 cm in diameter have a greater probability of being clear of lymph node involvement, are better differentiated, and have less perineural involvement than larger tumors [19].

Palliative surgery was traditionally considered a gold standard with the realization of biliary-digestive and gastro-jejunal anastomosis. In our study, 11% of patients had an internal bypass. Surgical palliation, reserved for patients already undergoing attempted surgical resection, and those with severe symptoms in whom stenting cannot be performed, is associated with a perioperative mortality rate of up to 14% [20]. Postoperative mortality rate in our study was 8%. Endoscopic stenting, an alternative to palliative surgery, is a well-tolerated and less invasive procedure. When comparing surgical bypass and endoscopic stenting, recurrent symptoms, such as jaundice or cholangitis, are significantly higher in patients who underwent stenting, whereas morbidity tended to be higher after palliative bypass [21]. Recently, endoscopic ultrasonography-guided gastroenterostomy showed efficacy similar to that of surgical bypass and duodenal stenting, with lower reintervention rates and reduced adverse events [22].

Neoadjuvant radiochemotherapy could be indicated in patients with borderline resectable pancreatic adenocarcinomas to increase the chances of complete R0 resection [23]. In our study, preoperative chemotherapy or radiochemotherapy was not correlated with overall survival. The PREOPANC randomized phase III trial showed that preoperative chemoradiotherapy was associated with better disease-free survival, lower rates of pathologic lymph nodes, perineural invasion, and venous invasion but no benefit for overall survival [24]. Consistent with the results of other studies, palliative radiotherapy was associated with prolonged survival in our study [25]. Palliative radiotherapy could also be an option for improving pain control in patients with locally advanced or metastatic pancreatic cancer [26].

Surgical resection in combination with systemic treatment remains the only chance of long-term survival or cure in patients with primary pancreatic cancer. After macroscopic complete resection for ductal adenocarcinoma of the pancreas, the adjuvant combination of gemcitabine and capecitabine or the FOLFIRINOX regimen led to improved overall survival [10,27,28]. Currently, many clinical trials seek to assess the efficacy of immunotherapeutic strategies in pancreatic cancer, but further studies are necessary to show practice-changing results [29]. In our study, the adjuvant and the palliative chemotherapy were associated with improved survival. Different chemotherapy regimens can add between 6 weeks and 11 months to the expected life span of a patient with advanced pancreatic cancer [30]. Besides potential sides effects of medical treatment, the dorsal/abdominal pain, obstructive jaundice, and gastric outlet obstruction, patients with advanced disease will spend about 10% of this limited time on outpatient health care [31].

Our work has several limitations. First, its retrospective design did not allow the collection of certain types of data such as performance status at diagnosis, possible changes in medication, nature and treatment of complications, chemotherapy regimens, radiotherapy doses, and adverse effects. However, our data were collected at several sites and describe the “real-life” management of those patients. Since the entire population of interest was included, center heterogeneity and selection biases (hepatobiliary vs. general surgeons, emergency vs. routine procedures, etc.) that affect traditional institution-based observational studies were minimized [32]. Second, the retrospective design of this study meant that we have limited data on the quality of life and palliative care of metastatic patients. Palliative care of metastatic patients will be addressed more thoroughly in a future work by using a prospective cohort, which is currently being recruited. Finally, our study included patients who underwent curative resection and patients who received palliative treatments. This allowed us to analyze the prognostic factors involved in the overall survival of patients with advanced disease at diagnosis and patients with recurrence after surgery.

## 5. Conclusions

The decision of treatment (surgery, chemotherapy, best supportive care) for patients with pancreatic adenocarcinoma should consider all the prognostic factors and life expectancy of each patient. In our population-based study, surgical resection is the only potentially curative treatment, but less than a quarter of patients were eligible. Our data suggest that chemotherapy (adjuvant or palliative) was associated with better outcomes. The risk factors and therapy modalities in pancreatic cancer patients deserve further study to improve the prognosis of this lethal disease.

## Figures and Tables

**Table 1:** Characteristics of patients (*n*, %).

Characteristics	Total *n* = 2117	Pancreatic Cancer Resection *n* = 293 (13.8%)	Palliative Treatment *n* = 1824 (86.2%)	*p*-Value
**Gender**				0.98
Male	1081 (51.1)	149 (50.85)	932 (51.09)	
Female	1036 (48.9)	144 (49.15)	892 (48.91)	
**Diagnostic Age (years)**	73 (IQR * 65.0–81.0)	69 (IQR 62.0–75.0)	75 (IQR 66.0–82.0)	<0.0001
**Primary tumor site**				<0.0001
Head	1120 (52.9)	208 (70.99)	912 (50.00)	
Neck	281 (13.3)	24 (8.19)	257 (14.09)	
Body	301 (14.2)	30 (10.24)	271 (14.86)	
Tail	53 (2.5)	6 (2.05)	47 (2.58)	
Diffuse lesions	362 (17.1)	25 (8.53)	337 (18.47)	
**Clinical manifestations**				
Jaundice	707 (33.39)	151 (51.53)	556 (30.48)	<0.0001
Weight loss	949 (44.8)	69 (23.54)	880 (48.24)	<0.0001
Abdominal pain	1016 (47.99)	113 (38.56)	903 (49.50)	<0.001
Unknown	195 (9.2)	40 (13.65)	155 (8.49)	

* IQR—interquartile range.

**Table 2:** Survival and prognostic factors (Cox regression analysis).

Characteristics	Univariate Analysis			Multivariate Analysis		
	HR ^1^	95% CI ^2^	*p*	HR ^1^	95% CI ^2^	*p*
**Age**	1.01	1.01 to 1.01	<0.0001	1.01	1.01 to 1.02	<0.0001
**Tumor location (tail)**	1.3	1.14 to 1.49	0.0001			
Surgical resection *	0.36	0.31 to 0.42	<0.0001	0.38	0.33 to 0.44	<0.0001
**Tumor size**	1.01	1.01 to 1.02	<0.0001			
**Well differentiated**	0.67	0.57 to 0.77	<0.0001			
**Moderately differentiated**	0.83	0.73 to 0.93	0.003			
**Lymphatic tumor emboli**	1.2	1.09 to 1.32	0.0001			
**Positive lymph nodes**	1.24	1.12 to 1.38	0.0001	1.24	1.08 to 1.35	<0.001
Absence of metastases **	0.65	0.59 to 0.72	<0.0001	0.66	0.59 to 0.75	<0.0001
**Postoperative chemotherapy**	0.36	0.22 to 0.60	0.0001	0.36	0.20 to 0.63	<0.001
**Palliative chemotherapy**	0.52	0.35 to 0.76	0.0009	0.63	0.42 to 0.93	0.02
**Palliative radiotherapy**	0.69	0.56 to 0.85	<0.0001	0.73	0.57 to 0.91	0.007

* Surgical resection of pancreatic cancer; ** Absence of metastases at diagnostic; ^1^ HR = hazard ratio, ^2^ CI = confidence interval.

## Data Availability

On request to corresponding author, upon reasonable request.
